# Limb Bone Structural Proportions and Locomotor Behavior in A.L. 288-1 ("Lucy")

**DOI:** 10.1371/journal.pone.0166095

**Published:** 2016-11-30

**Authors:** Christopher B. Ruff, M. Loring Burgess, Richard A. Ketcham, John Kappelman

**Affiliations:** 1 Center for Functional Anatomy and Evolution, Johns Hopkins University School of Medicine, Baltimore, Maryland, United States of America; 2 Department of Geological Sciences, The University of Texas Austin, Austin, Texas, United States of America; 3 Department of Anthropology, The University of Texas Austin, Austin, Texas, United States of America; University of Delaware, UNITED STATES

## Abstract

While there is broad agreement that early hominins practiced some form of terrestrial bipedality, there is also evidence that arboreal behavior remained a part of the locomotor repertoire in some taxa, and that bipedal locomotion may not have been identical to that of modern humans. It has been difficult to evaluate such evidence, however, because of the possibility that early hominins retained primitive traits (such as relatively long upper limbs) of little contemporaneous adaptive significance. Here we examine bone structural properties of the femur and humerus in the *Australopithecus afarensis* A.L. 288–1 ("Lucy", 3.2 Myr) that are known to be developmentally plastic, and compare them with other early hominins, modern humans, and modern chimpanzees. Cross-sectional images were obtained from micro-CT scans of the original specimens and used to derive section properties of the diaphyses, as well as superior and inferior cortical thicknesses of the femoral neck. A.L. 288–1 shows femoral/humeral diaphyseal strength proportions that are intermediate between those of modern humans and chimpanzees, indicating more mechanical loading of the forelimb than in modern humans, and by implication, a significant arboreal locomotor component. Several features of the proximal femur in A.L. 288–1 and other australopiths, including relative femoral head size, distribution of cortical bone in the femoral neck, and cross-sectional shape of the proximal shaft, support the inference of a bipedal gait pattern that differed slightly from that of modern humans, involving more lateral deviation of the body center of mass over the support limb, which would have entailed increased cost of terrestrial locomotion. There is also evidence consistent with increased muscular strength among australopiths in both the forelimb and hind limb, possibly reflecting metabolic trade-offs between muscle and brain development during hominin evolution. Together these findings imply significant differences in both locomotor behavior and ecology between australopiths and later *Homo*.

## Introduction

The acquisition of terrestrial bipedal locomotion was one of the defining events in hominin evolution [1–4]. Although there is anatomical evidence for some form of terrestrial bipedality among hominins at least 6 million years ago [5, 6], there is also accumulating evidence that the form of terrestrial bipedality practiced by some early hominins may have differed from that of modern humans, and that arboreal behavior continued to be an important component of the hominin locomotor repertoire for millions of years [5, 7–19].

There is continuing debate, however, over the functional significance of various postcranial morphological traits observed in early hominins, and whether they constitute primitive retentions of little adaptive importance or indicate major differences in actual locomotor behavior [4, 15, 20]. A key issue in such debates is the degree of developmental plasticity of different skeletal features. As noted by a number of authors, developmentally plastic traits should reflect, at least in part, the actual mechanical loading environment of the animal while it was alive, thus providing direct evidence of its behavior [4, 13, 21–26]. One such trait is the cross-sectional structure of long bone diaphyses, which is known from both experimental and observational studies to be responsive to changes in mechanical loadings during life [27–29]. Thus, for example, changes in inter-limb bone diaphyseal strength proportions accurately reflect changes in locomotor behavior during development in humans, gorillas, and chimpanzees [30–32]. Limb bone strength proportions in early *Homo erectus* (KNM-ER 1808 and KNM-WT 15000) are similar to those in modern humans [33], supporting other evidence for completely modern terrestrial locomotor behavior in this taxon [34]. In contrast, strength proportions in *H*. *habilis* (sensu stricto, OH 62) are more chimpanzee-like, suggesting a significant arboreal component in its locomotor repertoire [13].

To date, in part because of the fragmentary nature of most early hominin fossils, no similar analysis of an australopith individual has been carried out. Instead, analyses have focused on indirect assessments of limb bone proportions, through resampling techniques or comparisons between elements from different individuals, or between functionally disparate characteristics, such as articular and length proportions [17, 20, 35–37], which are difficult to interpret. Another issue with such analyses is that long bone length and articular size appear to be more genetically canalized than diaphyseal cross-sectional structure [31, 38–41], thus leading to familiar arguments regarding their effectiveness in reconstructing actual behavior during life [4, 20]. Diaphyseal external breadths or circumferences, included in some previous studies [37, 42], are also less effective than true cross-sectional properties in distinguishing between locomotor groups [13, 43].

**Fig 1:**
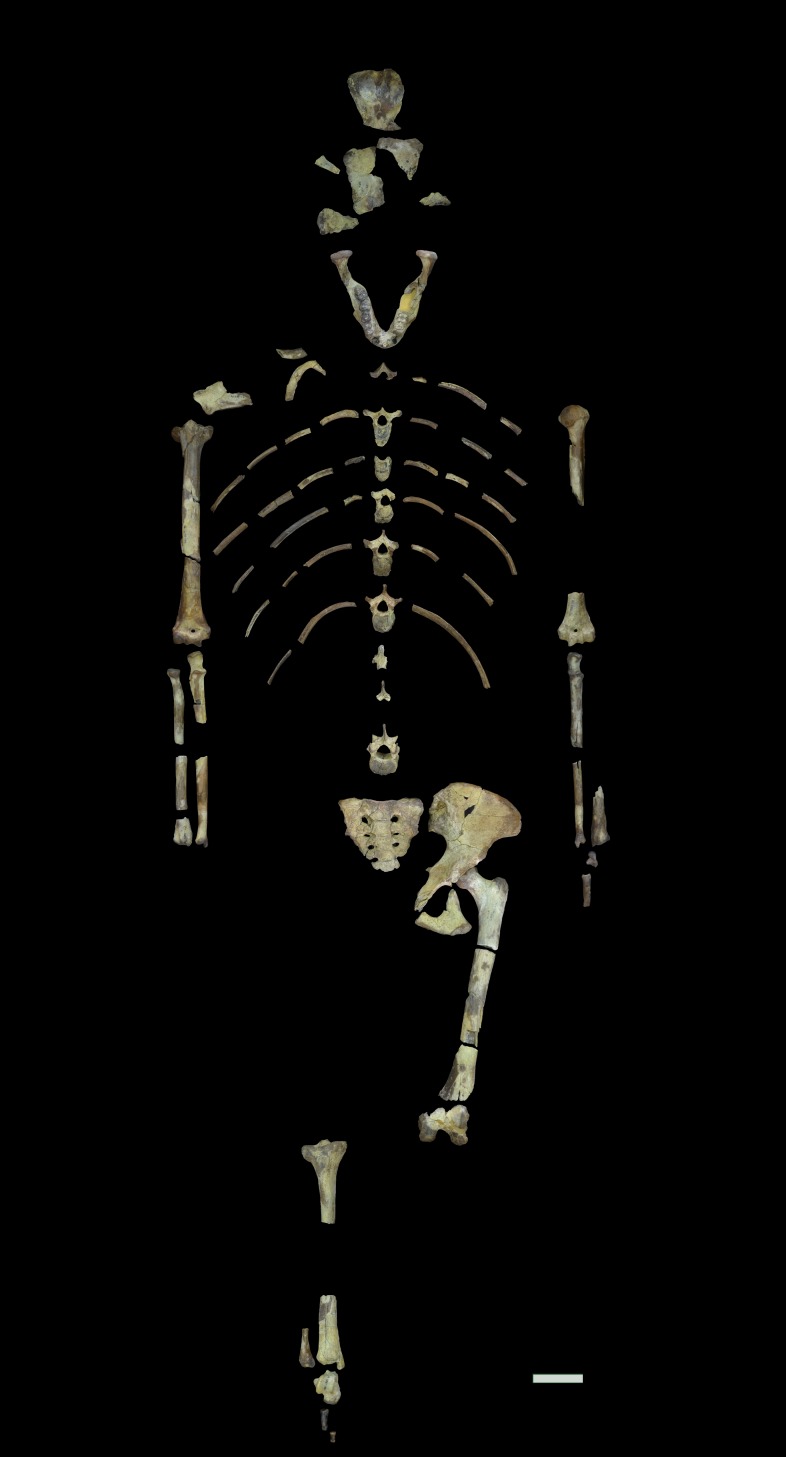
Composite photograph of original fossil specimens comprising A.L. 288–1. Scale bar is 50 mm.

The *Australopithecus afarensis* specimen A.L. 288–1 ("Lucy") is one of the very few early hominin (3.2 Myr) individuals to preserve relatively complete upper and lower limb bone elements [44] (Fig 1). A.L. 288–1 is a young adult female who had reached skeletal (all epiphyses fused) and dental (m3 erupted and in occlusion) maturity at death. It has long been recognized that her humerofemoral length proportion is intermediate between that of modern humans and chimpanzees, although this has led to different interpretations of her locomotor capabilities [45–49], and again is subject to the argument of primitive retention versus contemporary function [20]. Although diaphyseal cortices of the left femur and humerus of A.L. 288–1 are relatively well preserved (see below), no radiographic study of internal bone structure of these elements has been carried out to date (two earlier studies estimated some section properties from casts and photographs of natural breaks [50, 51]; these studies are discussed later in this paper). In this study, we use micro-CT to derive cross-sectional geometric properties of the humeral and femoral diaphyses and femoral neck of A.L. 288–1. Together with limb bone articular properties, these data are used to investigate inter- and intra-limb structural proportions of A.L. 288–1 in comparison to those of modern humans, chimpanzees, and other fossil hominin specimens, and to reconstruct locomotor behavior in early hominins.

We also use these data to address the general issue of relative muscular strength in early hominins, which has important implications for interpreting metabolic trade-offs, energy allocation, and ecology [52–56]. Because external articular size is less developmentally plastic than diaphyseal cross-sectional geometry [39, 41, 57], variation in diaphyseal strength to articular size should reflect to some extent relative muscular loadings during life (see S1 Text, S1 Fig). In addition, relative articular size is related in a long-term evolutionary timeframe to joint reaction force (JRF) and joint mobility [43, 58, 59]. These factors are also considered in our interpretations of variation in articular and shaft strength proportions in A.L. 288–1 and comparative samples. Our results indicate that A.L. 288–1, and australopiths in general, show differences in limb bone structural proportions from those of modern humans and *Homo erectus* that indicate significant differences in both locomotor behavior and relative muscularity.

## Materials and Methods

### Derivation of structural properties in A.L. 288–1

CT scans of the left and right humerus (A.L. 288-1r, s, m) and left femur (A.L. 288-1ap) of A.L. 288–1 were performed at the University of Texas High-Resolution X-ray CT Facility with procedures described in [60], using a FeinFocus FXE 225 kV X-ray source and image intensifier detector captured by a 1024x1024 CCD camera (original specimens are housed in the National Museum of Ethiopia, Addis Ababa, Ethiopia). Samples were held in place by custom foam mounts within Plexiglas containers, and X-ray signal was calibrated through empty containers. X-ray settings were 180 kV and 0.175–0.180 mA, with an estimated focal spot size of ~40 μm, and no beam filtration was used. Scanning parameters were optimized for each piece of the fossil based on size, and in some cases multiple pieces were scanned simultaneously. During each turntable rotation, 1200 views (projections) were acquired to obtain raw data for 25 slices. Raw data were reconstructed as 16-bit TIFF images. Beam hardening corrections were performed during reconstruction using polynomial linearization [61], with coefficients selected independently for each scan due to variations in mineralization. Ring artefacts were corrected either pre- or post-reconstruction [62]. Reconstruction scaling was reduced for pieces that had highly attenuating mineralization, probably oxides or sulfides, to avoid information loss from voxel saturation. Details on acquisition parameters, data voxel dimensions, and scaling and artefact processing parameters are given in S1 Table.

**Fig 2:**
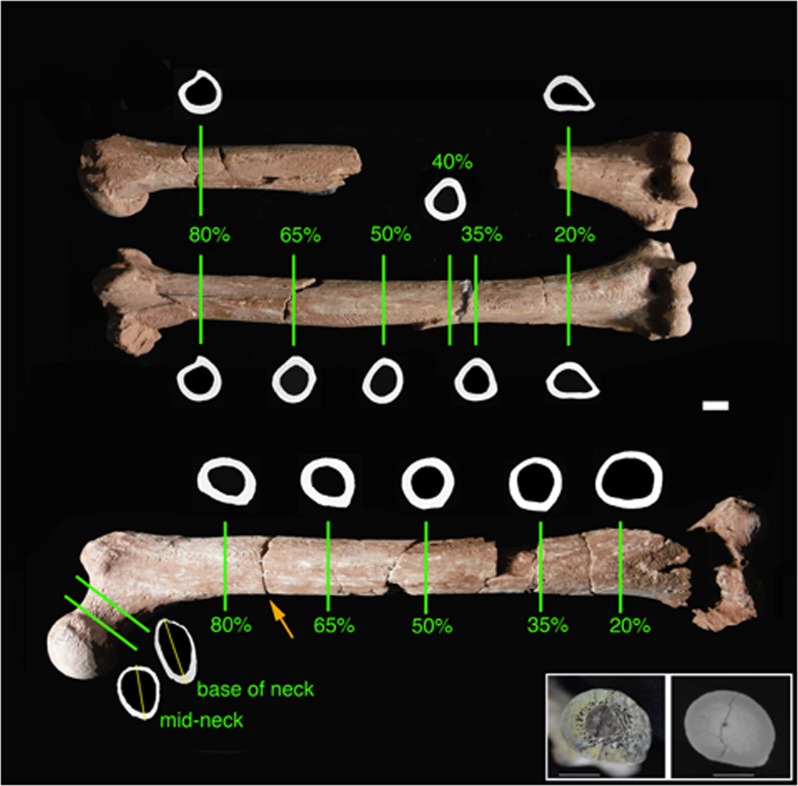
Section locations and cortical bone cross-sectional outlines for A.L. 288–1 humeri and femur, determined from CT scans. For diaphyseal sections, medial is to the left, anterior above; for femoral neck sections, anterior is to the left, superior above (medial and lateral orientations of (left) femur and left humerus reversed for consistency). Yellow lines through femoral neck sections indicate planes where superior and inferior cortical thicknesses were measured (as in [14]). Inset at lower right shows physical section (left) and CT image at an adjacent location (right) for a natural break in the femur at about 75% of length', indicated with an orange arrow in the main figure. Scale bars are 10 mm.

Scans were 3-D printed and measured to determine positions of comparative cross sections. The femur and humeri were oriented according to global anatomical axes, and bone lengths used for determination of cross section locations (length') measured as previously defined [43]. (Note that length' for the femur does not include the femoral head.) Reconstructed length' in the femur was 263 mm and in the humerus 236 mm. Cross sections were obtained at percentages of these lengths, at 20%, 35%, 40% (humerus only), 50%, 65%, and 80%, measured from the distal end [43], and through the mid- and base of the femoral neck, transverse to the neck axis [14]. Only the 20% and 80% sections were measurable for the left humerus. See Fig 2 for positioning of sections. Based on the most realistic reconstruction of the femoral condyles, the femur was oriented with a femoral neck anteversion angle of 22°, which is fairly high compared to some early *Homo* specimens [63], but similar to that estimated for the next most complete *Au*. *afarensis* femur, AL 827–1 (about 24°, CV Ward, pers. comm.). Data volumes were loaded into Avizo (FEI) for extraction of oriented sections. Scans not acquired in an orientation appropriate for a given anatomical comparison were obliquely re-sliced; angular departures of extracted slices from original data orientation are reported in the scanning parameters table (S1 Table).

Variations in data values among scans due to different acquisition and CT reconstruction and beam-hardening correction parameters necessitated renormalization to maximize consistency for measurements. Rescaling was done in IDL (Exelis Visual Information Solutions) on 16-bit data to align histogram peaks for mineralized cortical bone and air. The inset in Fig 2 illustrates the close correspondence between CT and physical sections at a natural transverse break in the femur about 75% of length from the distal end.

Images were then imported into Photoshop. Defects in periosteal and endosteal contours, i.e., cracks and chips, were repaired manually with reference to preserved cortical boundaries of the sections, as well as adjacent proximal and distal sections. In two cases some more major reconstruction was necessary. The lateral cortex of the right humerus (A.L. 288-1m) is broken and laterally displaced in the region of the 40% section (see Fig 2). The broken portion was manually extracted in the image and moved posteriorly and medially until periosteal and endosteal contours were aligned with the main piece. Aside from displacement, there was no apparent distortion in the broken portion and the final section contours appear normal (Fig 2). However, because of the greater reconstruction necessary, and thus potential for error, the humeral 35% section, which required minimal repairs, was used in comparative analyses rather than the 40% section, which has been included in some previous studies [13, 33]. The lateral supracondylar ridge in the region of the 20% section of the left humerus (A.L. 288-1s) is largely sheared off (also see [44]). The periosteal contour of this section was reconstructed by reversing and superimposing the analogous contour of the right humerus 20% section, which was undamaged. Slight alterations in reconstruction had minimal effects on cross-sectional properties (also see below regarding assessments of humeral bilateral asymmetry). The 35% section of the femur was taken slightly distal to its actual location to avoid an area where the cortex is missing (Fig 2).

In general, aside from obvious identifiable defects, periosteal surfaces appear relatively well preserved and undistorted, as do internal regions where trabecular bone is present. Endosteal boundaries were distinguished visually using greyscale contrast between the cortex and matrix in the medullary cavity, which in most cases was easily identified (see inset in Fig 2). Repeated reconstructions of the medullary cavity yielded very similar results in terms of geometry and section properties. It should also be noted that variation in endosteal dimensions has much less effect on the primary structural characteristics evaluated here, i.e., section moduli, than variation in periosteal dimensions [64].

Cross-sectional diaphyseal properties were calculated from images using NIH ImageJ and the Momentmacro macro (http://www.hopkinsmedicine.org/fae/mmacro.html). Unlike several past analyses [13, 33, 43, 65], true section moduli, utilizing maximum distances from the section neutral axis or centroid, were used here for the primary analyses of femoral/humeral strength and strengths relative to articular dimensions. This entailed remeasurement or recalculation of properties for some of the comparative samples, as described further below.

Section properties for all diaphyseal locations in A.L. 288–1 are given in Table 1. As in several other studies [13, 30, 31, 33], inter-limb strength comparisons here focus on the polar section modulus (Z_p_), which is an index of torsional and average bending strength. This is the most relevant structural parameter here, given the preponderance of bending and torsional loads in limb bones during active locomotion (e.g., [66]). Femoral 50% and humeral 35% sections are used in inter-limb and limb strength/articular size comparative analyses. Anteroposterior (A-P) and mediolateral (M-L) strengths are also assessed in the most proximal (80%) femoral diaphyseal section, since it has been demonstrated that cross-sectional shape here is informative regarding hip loadings [65].

**Table 1:** Cross-sectional diaphyseal properties of A.L. 288–1.

Element	Section	TA	CA	%CA	Ix	Iy	Imax	Imin	J	Zx	Zy	Zp
Rt. Humerus	20	214.8	112.9	52.6	2308	3820	3941	2187	6128	281	341	525
Rt. Humerus	35	230.0	136.5	59.4	3648	3486	3654	3479	7134	384	394	741
Rt. Humerus	40	221.3	134.7	60.9	3382	3292	3603	3071	6674	373	372	727
Rt. Humerus	50	227.6	126.6	55.6	3761	2972	3927	2806	6733	389	349	695
Rt. Humerus	65	240.9	139.8	58.0	4159	3557	4424	3291	7715	441	398	803
Rt. Humerus	80	215.6	120.0	55.7	2993	3035	3079	2949	6028	316	332	636
Lt. Humerus	20	213.4	117.4	55.0	2364	3753	3875	2242	6117	301	341	524
Lt. Humerus	80	208.5	108.0	51.8	2689	2646	2745	2591	5336	274	303	542
Femur	20	479.5	206.3	43.0	10897	13783	13895	10786	24681	919	969	1728
Femur	35	358.1	176.0	49.1	7514	7535	7825	7224	15049	665	706	1312
Femur	50	330.1	197.6	59.9	7221	7327	7536	7011	14548	688	691	1350
Femur	65	329.0	200.5	60.9	6798	8015	8447	6366	14813	694	719	1246
Femur	80	332.1	197.8	59.5	5948	9399	9864	5483	15347	616	792	1290

See Fig 2 for locations of sections. TA: total subperiosteal area (mm^2^); CA: cortical area (mm^2^); %CA: (CA/TA)*100; Ix, Iy: second moments of area (SMA, in mm^4^) in AP and ML planes; Imax, Imin: maximum and minumum SMA; J: polar SMA; Zx, Zy, Zp: AP,ML, and polar section moduli (mm^3^).

Superoinferior breadth of the femoral head (28.6 mm), and M-L breadths of the proximal tibial (50.2 mm) and distal humeral (30.1 mm) articular surfaces of A.L. 288–1 were derived from values reported in the literature [44, 67]. Measurement definitions follow [43]. Femoral neck superior and inferior cortical thicknesses were measured from CT images as described previously [14] along axes illustrated in Fig 2. Measurement of femoral biomechanical neck length followed [65].

### Comparative samples

The modern human sample used in comparisons of femoral/humeral strength and strength/articular proportions was derived from an ongoing study of Upper Paleolithic to 20th century European skeletal samples [68, 69]. Sample sizes vary between 838 and 1756, depending on the comparison (S2 Table). Proximal femoral (80% location) sections were not included in that study, so for those comparisons of that section modern human data were obtained from a previous study of Pecos Pueblo Native American and recent East African samples (combined n = 100) [65]. Following previous studies [43, 65, 70], A-P and M-L section moduli for the 80% section in these samples were derived from A-P and M-L second moments of area (SMA) as SMA^.73^. Cross-sectional diaphyseal data were obtained using a number of techniques, including direct sectioning [71], radiography and external molding [72], and CT scanning. All individuals were adult, with fused long bone epiphyses. Because of known bilateral asymmetry in modern human humeri and possible bilateral asymmetry in the humeri of A.L. 288–1 (see below and S5 Table), all modern humeri were sampled on the same, i.e., right side used in A.L. 288–1 strength comparisons. Both right and left femora were sampled.

The chimpanzee comparative sample was derived from a previous [43] and ongoing [73] study of long bone structural variation in *Pan*. The sample includes three subspecies of common chimpanzees (*P*.*t*. *schweinfurthii*, *P*.*t*. *troglodytes*, *P*.*t*. *verus)* and bonobos (*P*. *paniscus*, about 20% of the total sample). Sample sizes vary between 93 and 98, depending on the comparison (S2 Table), with 23 individuals derived from the previous study and 70–74 from the new study. All individuals were wild collected and adult. Cross-sectional data were obtained using CT scanning. The previously collected [43] data did not include true section moduli (calculated using maximum radii). For the femoral 50% section, where diaphyseal breadths were available, Z_p_ (polar section modulus) was calculated from J (polar SMA) incorporating diaphyseal breadths, as described previously [43], but these were then adjusted using the relationship between these approximate Z_p_ values and true Z_p_ values in the more recently measured sample (r = .985, %SEE = 3.6%). Only humeral 40%, not 35% section data were obtained for the new sample, while both sections were measured in the old sample. A conversion equation derived from the old sample was used to calculate J values for the 35% section from 40% section values in the new sample (r = .997, %SEE = 2.3%). All J values were then converted to Z_p_ values as J^.75^ (based on scaling relationships for the 40% section in the new sample). The mean difference between estimated Z_p_ values at the 35% and true Z_p_ values at the 40% sections of the humerus in the new sample is less than 2%; i.e., the exact placement of this section and method of calculation make very little difference in comparisons. Both right and left sides were included, although the majority of specimens were from the right side.

Structural properties of other fossil hominins were obtained from previously published data [33, 65, 74–79] and new data derived for this study (Table 2). Given the absence of craniofacial associations for many of these specimens, taxonomic assignments are often not certain. The least secure attributions are indicated with question marks. For convenience in some subsequent presentation and discussion of results, fossil *Homo* specimens may be referred to simply as "early *Homo* ", to distinguish them from modern humans and australopiths. Public repositories of specimens are listed in Table 2.

**Table 2:** Comparative fossil specimens.

Taxon	Specimen	Date (Myr)	Graph No.	F50Zp	H35Zp	FHDSI	TPLML	HDARTML	F80ZX	F80Zy	Source	Repository
Australopithecus africanus	Stw 99	2.6	1	2499		39.1			1755	2257	1,11	1
Australopithecus africanus	Stw 431	2.6	2		1279	42.0		41.3			1,11	1
Paranthropus boisei?	KNM-ER 739	1.5	3		2217			45.6			1	2
Homo sp.	KNM-ER 1472	2.0	4	2395		40.3	64.5*		1040	1794	1,2	2
Homo sp.	KNM-ER 1481	1.9	5	1918		43.2	69.3*		1180	1800	1,2	2
Homo erectus	KNM-ER 1808	1.6	6	3216	884				1603	2442	1,3	2
Homo erectus	KNM-WT 15000	1.5	7	1828	763	44.9	66.8*	38.2			1,3	2
Homo erectus?	Gombore MK3	>1.4	8		1770			55.2			4,5	3
Homo antecessor	ATD6-148	0.9	9		900			44.6*			5,6	4
Homo heidelbergensis	Atap. SH-XV	0.6	10		1041			45.0			5,6	4
Homo heidelbergensis	Atap. SH-III	0.6	11		1510			47.9			5,7	4
Paranthropus robustus	SK 82	1.7	12						1461	1911	8	5
Paranthropus robustus	SK 97	1.7	13						1441	2023	8	5
Paranthropus boisei	KNM-ER 1500	1.9	14						921	1095	2	2
Paranthropus boisei	OH 80	1.3	15						1682	1965	9	6
Paranthropus boisei?	KNM-ER 993	1.5	16						1617	2128	2	2
Paranthropus boisei?	KNM-ER 1463	1.4	17						1053	1096	2	2
Paranthropus boisei?	KNM-ER 1503	1.9	18						1266	1631	2	2
Paranthropus boisei?	KNM-ER 815	1.8	19						737	1053	2	2
Paranthropus boisei?	KNM-ER 738	1.9	20						1008	1323	2	2
Paranthropus boisei?	KNM-ER 1465	1.5	21						1576	1980	2	2
Paranthropus boisei?	OH 20	1.7	22						1329	1802	2	6
Homo erectus	KNM-ER 737	1.6	23						1814	2905	2	2
Homo erectus	KNM-ER 803	1.5	24						1897	2593	2	2
Homo erectus	Kresna 11	>0.9	25						1696	2487	10	7
Homo erectus	OH 28	0.7	26						1521	2665	2	6

**Fig 3:**
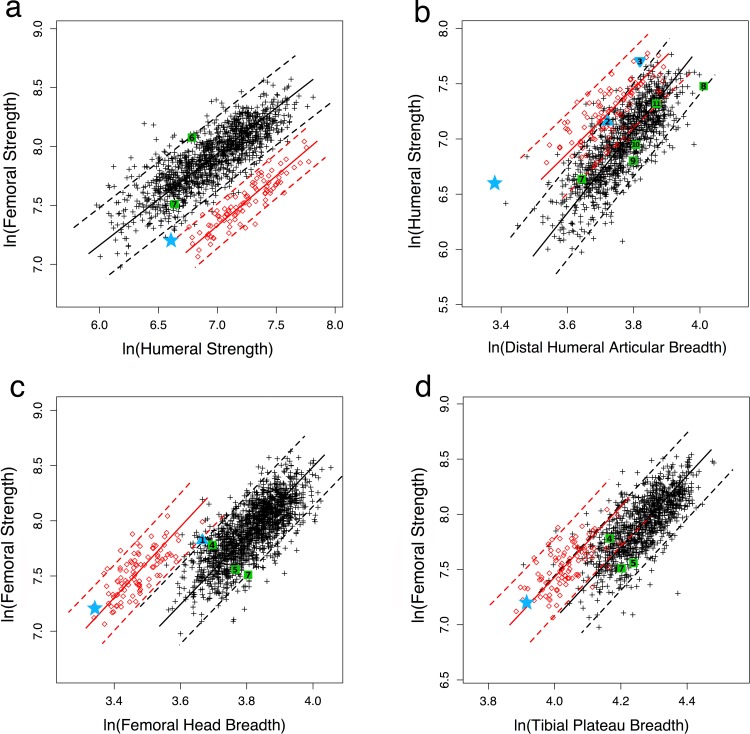
Overall diaphyseal strength (polar section modulus) proportions in A.L. 288–1 (blue star), other australopiths (blue triangles), early *Homo* (green squares), modern humans (black crosses), and chimpanzees (red circles). Femoral sections taken at 50% and humeral sections at 35% of length, measured from the distal end. Reduced major axis regression lines and 95% prediction intervals plotted for modern humans and chimpanzees. a) femoral/humeral strength, b) humeral strength/humeral distal articular breadth, c) femoral strength/femoral head breadth, d) femoral strength/proximal tibial articular breadth. Numbers refer to individual fossils in Table 2 (1: Stw 99; 2: Stw 431; 3: KNM-ER 739; 4: KNM-ER 1472; 5: KNM-ER 1481; 6: KNM-ER 1808; 7: KNM-WT 15000; 8: Gombore MK3; 9: ATD6-148; 10: Atap. SH-XV; 11: Atap. SH-III).

**Fig 4:**
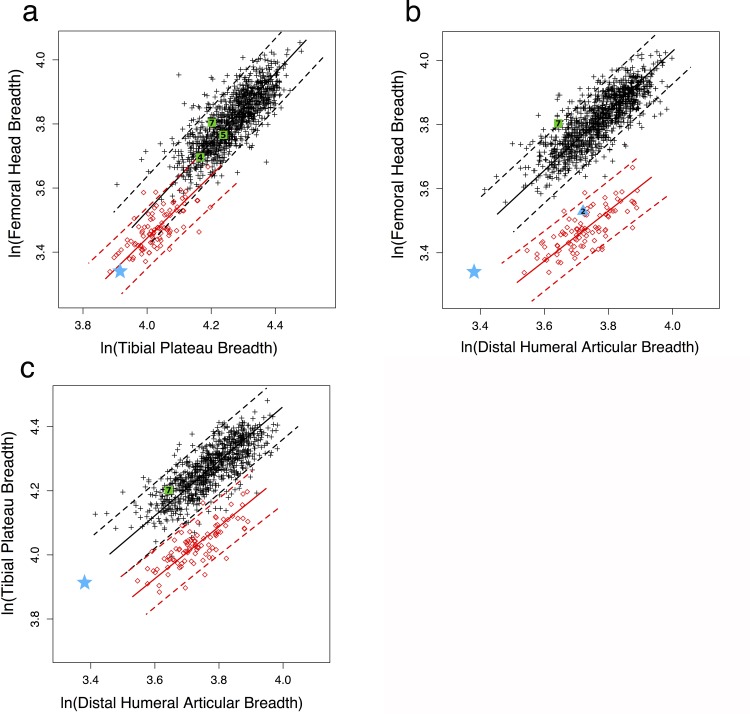
Articular breadth proportions in A.L. 288–1 (blue star), other australopiths (blue triangles), early *Homo* (green squares), modern humans (black crosses), and chimpanzees (red circles). Reduced major axis regression lines and 95% prediction intervals plotted for modern humans and chimpanzees. a) femoral head/proximal tibia, b) femoral head/distal humerus, c) proximal tibia/distal humerus. Numbers refer to individual fossils in Table 2 (2: Stw 431; 4: KNM-ER 1472; 5: KNM-ER 1481; 7: KNM-WT 15000).

**Fig 5:**
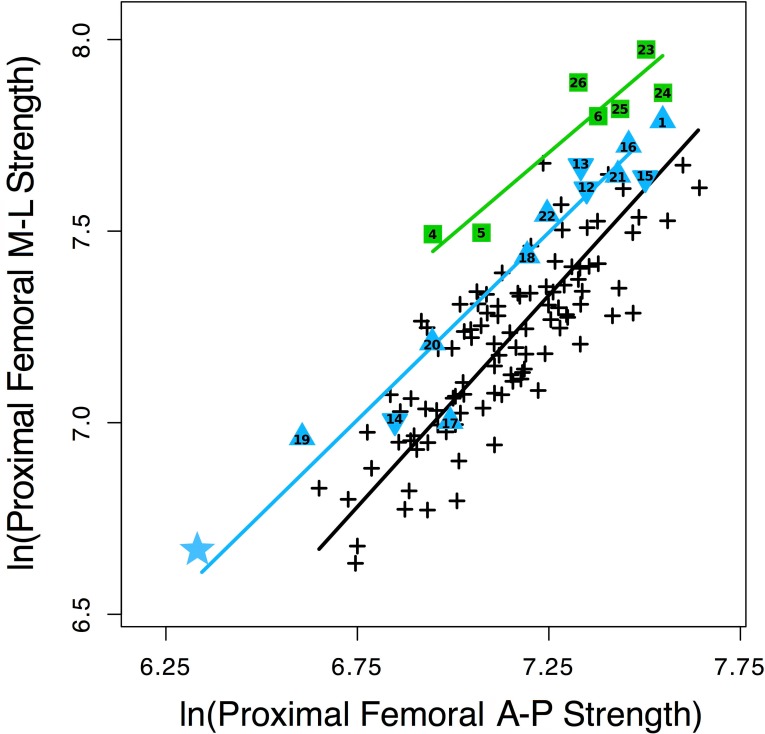
Mediolateral/anteroposterior bending strength (section moduli) proportions of the femoral 80% (subtrochanteric) section, in A.L. 288–1 (blue star), other australopiths (blue triangles), early *Homo* (green squares), and modern humans (black crosses), with reduced major axis regression lines through early *Homo*, australopiths, and modern humans. See Table 2 for identification of individual fossil specimens.

Graph numbers refer to Figs 3, 4 and 5.

*Estimate—see Materials and Methods.

F50Zp: femoral 50% polar section modulus; H35Zp: humeral 35% polar section modulus; FHSI: femoral head superoinferior breadth; TPLML: proximal tibial ML articular breadth; HDARTML: distal humeral ML articular breadth; F80Zx, Zy: femoral 80% AP and ML section moduli (derived as SMA^.73^—see Materials and Methods).

Sources: 1) Present study; 2) Ruff, 1995 [65]; 3) Ruff, 2008 [33]; 4) Di Vincenzo et al., 2015 [76]; 5) L. Rodriguez, pers. comm.; 6) Bermudez de Castro et al., 2012 [74]; 7) Carretero et al., 1997 [75]; 8) Ruff et al., 1999 [79]; 9) Dominguez-Rodrigo et al., 2013 [77]; 10) Puymerail et al., 2012 [78]; Ruff et al., in press [80].

Repositories of fossil specimens: 1) University of the Witwatersrand, Johannesburg, South Africa; 2) National Museums of Kenya, Nairobi, Kenya; 3) National Museum of Ethiopia, Addis Ababa, Ethiopia; 4) Museo de Burgos, Burgos, Spain; 5) Transvaal Museum, Pretoria, South Africa; 6) National Museum of Tanzania, Dar es Salaam, Tanzania; 7) Archaeological Service of Yogyakarta, Indonesia.

New cross-sectional data for Stw 99 and 431, and KNM-ER 739 were derived from CT scans using previously described procedures [14, 33, 80]. Several previously analyzed specimens (KNM-ER 1472, 1481, and 1808, KNM-WT 15000) were reanalyzed using the newer software employed for A.L. 288–1 (see above) to obtain true Z_p_ values; thus, these vary somewhat from those reported earlier for these specimens [33]. Also, values for the left femur of KNM-WT 15000, rather than an average of right and left side values as reported previously [33], were used here because articular breadths could be measured on the left side. Because data for only the humeral 40% section were available for KNM-ER 1808 and KNM-WT 15000, 35% Z_p_ values were derived from 40% values using the average ratio of the two in the Pecos Pueblo sample; as with chimpanzees (see above), values are very similar at the two locations (H35 Z_p_ /H40 Z_p_ = 0.975). Given known large changes in femoral diaphyseal cross-sectional shape during ontogeny [64, 81], KNM-WT 15000 is not included in analyses of proximal femoral shape. Because only A-P and M-L SMAs for the femoral 80% section were available for the extant and some of the fossil comparative specimens, for comparative analyses all Z_x_ and Z_y_ values for this section were derived as SMA^.73^, as described above.

Distal humeral M-L articular breadth of ATD6-148 is the mean of estimates based on humeral biepicondylar breadth reported in [74]. Proximal tibial M-L articular breadth for KNM-ER 1472, 1481, and KNM-WT 15000 was calculated from distal femoral articular breadth [82] using a regression equation derived from the modern European sample (n = 1023, r = .892, %SEE = 3.6%). The estimate for KNM-ER 1481 (69.3 mm) is slightly smaller than that previously estimated based on reconstruction of the less well preserved proximal tibia (72.4 mm) [82]. Distal femoral articular breadth for KNM-WT 15000 (65 mm) was measured on the original specimen without correction for probable M-L compression of the epiphysis [73]; it is likely that true articular breadths of the distal femur and proximal tibia of this specimen were somewhat wider.

Femoral length' [43] and biomechanical neck length [65] for three femora from Sima de los Huesos, Sierra de Atapuerca (S4 Table) were measured from A-P photographs in Figure 2 of [83]. Measurements of other specimens were derived from previous studies [63, 65] (see S4 Table).

### Statistical methods

Reduced major axis (RMA) analyses were carried out in R using the Imodel2 and smatr packages [84, 85]. For comparisons between samples where slopes were not equivalent, the nonparametric Quick Test [86] was used for elevational tests. Relative deviations of fossil specimens from each modern comparative sample regression line are expressed in SEE (Standard Error of Estimate) units, as calculated for RMA regressions [13, 87]. For graphing purposes, 95% prediction intervals (PI) were calculated using this SEE in the following equation:

$$95\%\;PI={t\;}_{0.05(2)}∙\;\sqrt{\left(2∙{SEE}^{2})∙\;\mathrm{RMA slope}\;[1+\frac{1}{n}+\frac{{\left(x_{i}-\overset{-}{x}\right)}^{2}}{\Sigma x^{2}}\right]}$$

Humeral bilateral asymmetry was assessed using the formula ((R-L)/((R+L)/2))*100. Because there is some uncertainty in the exact concordance of section locations in the proximal (80%) section of the right and left humeri of A.L. 288–1 (due to the disjunction between proximal and distal portions on the left side, and crushing of the proximal end on the right side), bilateral asymmetry was also assessed for combinations of section locations ±1–3 mm proximal and distal to the best estimate of the true section location, as available given preservation on each side. Results were similar to those obtained using the best estimate (S5 Table).

## Results

As predicted given their locomotor differences and results of previous studies based on smaller samples [13, 30], modern humans have stronger femora relative to humeri than chimpanzees, with non-overlapping 95% prediction intervals (PI) (Fig 3A, S3 Table). A.L. 288–1 falls below the 95% PI for modern humans and just above the upper 95% PI for chimpanzees, but closer to the average strength proportions of chimpanzees (S3 Table). As shown previously [33], two early (1.6–1.7 Myr) *Homo* specimens, KNM-WT 15000 and KNM-ER 1808, have femoral to humeral strength proportions well within the range of modern humans (Fig 3A; S3 Table). The lower value for KNM-WT 15000 is attributable to his juvenile status, since femoral/humeral strength increases during development in humans and does not reach fully adult proportions until late adolescence [30, 33].

Also as predicted, given their relatively greater muscular strength [52, 56] (S2 Text), chimpanzees have greater diaphyseal strength relative to articular size than modern humans in both the femur and humerus (Fig 3B–3D, S2 Table), although 95% PI's overlap, except for femoral strength relative to femoral head breadth. A.L. 288–1 has an exceptionally strong humerus relative to humeral distal articular breadth, falling close to the upper 95% PI for chimpanzees and far outside the modern human range (Fig 3B). Two specimens attributed to *Au*. *africanus* (Stw 431, 2.6 Myr) and possibly *Paranthropus boisei* (KNM-ER 739, 1.5 Myr) also have relatively strong humeral diaphyses, falling much closer to average chimpanzee proportions and very near or above the 95% PI for modern humans (Fig 3B, S3 Table). In contrast, five early (0.6–1.5 Myr) *Homo* or putatively *Homo* specimens all have humeral strength to articular proportions much closer to those of modern humans than chimpanzees. The same general observations hold true for femoral strength to articular proportions: A.L. 288–1 has a strong femoral diaphysis relative to articular breadths of the femoral head and proximal tibia, similar to chimpanzees and outside the 95% CI of modern humans, while early *Homo* specimens fall closer to modern humans (Fig 3C and 3D; S3 Table). The particularly low value for KNM-WT 15000 in femoral shaft strength/head breadth in Fig 3C is again attributable to his developmental status [41]. The one other australopith specimen possible to include here, Stw 99 (*Au*. *africanus*), is intermediate between humans and chimpanzees in femoral shaft strength/head breadth.

The greater overlap between modern humans and chimpanzees in femoral diaphyseal strength relative to knee breadth than to femoral head breadth (Fig 3C and 3D) implies that modern humans have relatively large femoral heads compared to other lower limb joints. This conclusion is confirmed in Fig 4A, where modern humans overlap with, but are statistically distinct from chimpanzees in femoral head/knee breadth (S2 Table). Three early (1.5–2.0 Myr) *Homo* specimens all fall well within the modern human distribution for this articular proportion, while A.L. 288–1 is much more similar to chimpanzees (Fig 4A, S3 Table). When femoral head breadth is compared to distal humeral breadth (Fig 4B), modern humans and chimpanzees are quite distinct, with one early *Homo* specimen (KNM-WT 15000) falling at the upper edge of the human distribution (possibly as a result of his juvenile status). A.L. 288–1 falls between the 95% PIs of humans and chimpanzees, although slightly closer to modern humans, while the *Au*. *africanus* Stw 431 falls closer to chimpanzees (S3 Table). However, A.L. 288–1 has knee to distal humeral articular breadth proportions much more similar to those of modern humans than chimpanzees, as does KNM-WT 15000 (Fig 4C, S3 Table).

**Fig 6:**
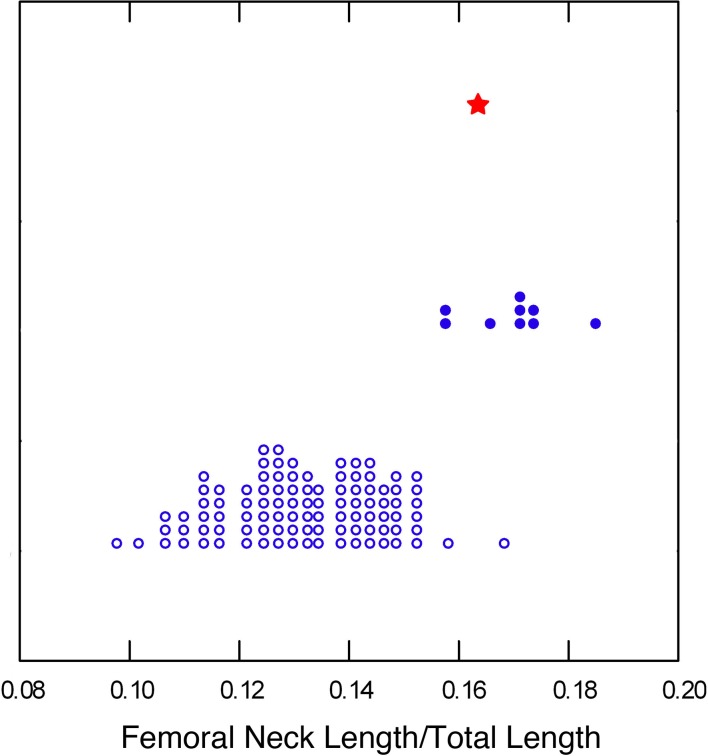
Femoral biomechanical neck length [65] relative to total femoral length (length'—see [43]) in modern humans (open circles), early *Homo* (closed circles), and A.L. 288–1 (star). Modern human data from Ref. [65]; see S4 Table for sources of early *Homo* data.

Together these observations indicate that when compared to modern humans or earlier *Homo*, A.L. 288–1 has a small femoral head, not only relative to diaphyseal strength but also to other limb articulations, including the knee. This suggests a specific reduction in relative hip joint reaction force (JRF) in A.L. 288–1 compared to *Homo*. Analyses of other proximal femoral structures provide further support for this inference. Both australopiths and early *Homo* have increased M-L relative to A-P strength of the proximal femoral diaphysis compared to modern humans (Fig 5). This difference is likely a result of the wide biacetabular breadth and relatively long femoral neck that characterizes all early hominins [14] (Fig 6, S4 Table), both of which would be expected to increase M-L bending loads in the proximal femur [65] (S3 Text). However, australopiths, including A.L. 288–1, have significantly less M-L strengthening of this region than early *Homo* (Fig 5; S2 Table). This observation runs counter to biomechanical predictions (S2 Fig) and is consistent with a reduction in hip JRF relative to that in early *Homo* or modern humans [51] (S3 Text).

**Fig 7:**
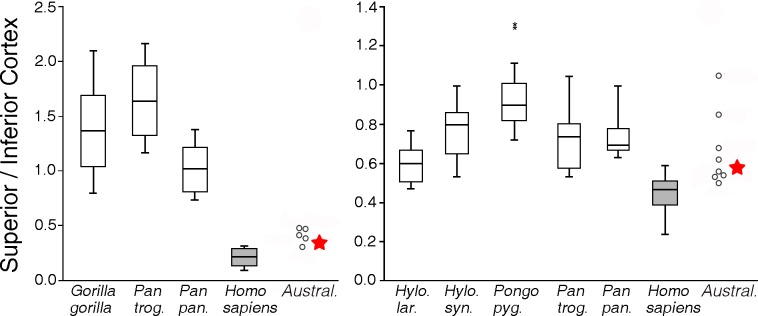
Superior/inferior femoral neck cortical breadths at: a) base of the neck and b) mid-neck (see Fig 2) in A.L. 288–1 (star), other australopiths (circles), and modern hominoids; comparative data from [14].

Internal femoral neck structure is also related to variation in hip joint loading. Modern humans have relatively thinner superior and thicker inferior cortices in the femoral neck than nonhuman hominoids, which has been explained as a consequence of the hip abductor mechanism in humans that maintains balance during bipedal walking, compared to more generalized loadings of the femoral neck in apes [14, 88]. Together with other australopiths, A.L. 288–1 has femoral neck cortical breadth ratios that generally fall closer to those of modern humans but are slightly higher, i.e., more ape-like (Fig 7). This pattern, and A.L. 288–1's general cross-sectional neck shape [14], imply a more vertically oriented hip JRF than in modern humans, which increases bending of the neck and tensile stresses on the superior cortex [14]. This neck morphology also has implications regarding possible gait differences (see Discussion).

Proximally (in the 80% section), A.L. 288–1's right humerus is significantly (10–15%) stronger than her left humerus (Table 1, S5 Table), suggesting that she was possibly right-handed. However, her distal (20%) humeral sections are almost symmetric. It is possible that the proximal asymmetry is related to greater muscular development in the right shoulder region; however, more information is needed on the relative developmental plasticity of different regions of the humerus to interpret these results in behavioral terms.

## Discussion

It is clear that A.L. 288–1 and australopiths in general show many postcranial adaptations to terrestrial bipedality and probably walked in a basically human-like manner when on the ground [4, 26, 44, 88–92]. The results of the present study reinforce this view. A.L. 288–1's knee to elbow size proportion is distinctly human and non-ape-like, indicating an adaptation to greater loading of the hind limb than in apes. Her femoral neck structure implies a hip abductor mechanism during bipedal gait that was broadly similar to that in modern humans.

However, we found that A.L. 288–1 also exhibits morphological features that imply substantial differences in locomotor behavior from that in modern humans or early *Homo*. Lucy’s femoral/humeral diaphyseal strength proportion indicates greater muscular loading of her upper limb relative to her lower limb than is characteristic of either modern humans or *Homo erectus*, and more similar to that of chimpanzees. While other behavioral explanations are conceivable (such as increased upper limb use related to food procurement or defense), given the range of morphological evidence throughout her skeleton that is consistent with greater arboreality [10, 19, 93], the most likely explanation is that Lucy climbed trees with a greater reliance on her upper extremity much more frequently than modern humans or early *Homo* (with the exception of *H*. *habilis sensu stricto* [13]). It is even possible that adaptations for terrestrial bipedality, such as restructuring the foot in *Au*. *afarensis* [4], necessitated greater compensatory muscular force in the upper limb to facilitate frequent climbing, which could account in part for her relatively increased humeral strength. The developmental plasticity of long bone diaphyseal structure negates the argument that this proportional difference in strength could be just a primitive retention from a more arboreal ancestor, and the degree of adaptation observed argues against its being an infrequent and minor component of the locomotor repertoire. These results also support the view that other potentially more genetically constrained morphological features observed in *Au*. *afarensis* that could facilitate arboreal behavior, such as longer and more curved toes and fingers, a higher intermembral index, and a more cranially oriented glenoid fossa [10, 18, 19, 46], were maintained by stabilizing selection for arboreality rather than simply not being selected against [4].

Our interpretation of the functional significance of inter-limb strength proportions is supported not only by direct experimental evidence for bone developmental plasticity in general [27], but also by comparisons of limb bone strength proportions between and within hominoids in relation to locomotor behavior. Among great apes, forelimb to hind limb strength proportions parallel the degree of arboreality, from orangutans to chimpanzees to Western lowland gorillas to mountain gorillas, the most terrestrial nonhuman hominoid [43, 94]. Furthermore, within both mountain gorillas and common chimpanzees, ontogenetic declines in arboreal locomotion are associated with declines in forelimb to hind limb strength [31, 32]. It should be noted that these trends are not simply a function of differences or changes in overall limb bone size, e.g., length, as lowland and mountain gorillas have similar limb length proportions but different strength proportions, and ontogenetic changes in limb length proportions in mountain gorillas do not parallel those in strength proportions [31]. The same is true for growing humans, who show much greater increases in femoral/humeral strength proportions than in length proportions, including a sharp reversal in strength proportions at the initiation of walking that has no counterpart in length changes [30, 95]. Infant (pre-walking) humans are in fact similar to quadrupedal primates in limb bone strength proportions, even though the hind limb already shows evidence of relative elongation, and only develop typically human strength proportions with the increased mechanical loading of the lower limb generated by bipedal gait [30]. Another interesting "natural experiment" in limb strength proportions among humans is provided by gymnasts, who habitually load their upper limbs at several times body mass during practice and performance [96], and show much greater increases in upper limb bone strength than in lower limb bone strength over non-gymnast controls [96, 97]. Exactly how upper limb bone loading is increased during arboreal locomotion among hominoids has not yet been quantified, although it seems probable that climbing and forelimb suspensory behavior produce relatively large mechanical loads on the forelimb bones. Although the forelimb is also loaded during terrestrial quadrupedal locomotion in nonhuman hominoids, peak vertical forces are much higher on the hindlimb [98].

A relatively longer humerus and shorter femur in A.L. 288–1 compared to modern humans [45–49] could also theoretically contribute to higher bending loads in the humerus in A.L. 288–1, by increasing bending moment arms. However, limb muscle moment arms in chimpanzees and modern humans, scaled for body size, are not systematically different and do not covary consistently with limb length differences [99]. Also, as discussed above, ontogenetic changes in bone length proportions are not paralleled by similar changes in bone strength proportions. Thus, it seems unlikely that limb length differences alone could account for proportional differences in strength between A.L. 288–1 and modern humans.

Other purportedly developmentally plastic traits such as phalangeal curvature have also been argued to support the argument for more arboreal behavior in australopiths and other early hominins [5, 18, 19, 77, 100]. However, it should be noted that the inference of developmental plasticity of phalangeal curvature is based only on comparative ontogenetic studies [21, 101, 102], and in addition to possible direct mechanical effects there is also likely a strong genetic component to curvature [102]. There are also undoubtedly genetic effects on long bone cross-sectional geometry (e.g., [103, 104]); however, there is also abundant experimental evidence that long bone strength is modified by behavioral use during life [27]. It should also be noted that genetic differences in skeletal mechanosensitivity to applied loadings, demonstrated in some modern inbred strains (e.g., [105]), cannot account for inter-limb proportional differences, since skeletal elements from the same individual are being compared. Thus, the present results provide the strongest evidence to date for increased mechanical loading of the upper relative to the lower limb in an australopith, which implies a significant difference in its locomotor repertoire. While direct femoral to humeral strength comparisons are not possible in other associated specimens, the very strong upper limb bones characteristic of australopiths generally ([77, 106, 107], and present study) are also consistent with this interpretation.

OH 62, attributed to *H*. *habilis* (sensu stricto) and dated to about 1.8 Myr [108], also shows increased upper relative to lower limb bone strength compared to modern humans [13]. Although not included in the present study because of uncertainties regarding the exact placement of available cross sections in OH 62, for the most reasonable reconstructions it clearly is more similar to chimpanzees in strength proportions, falling in a position similar to that of A.L. 288–1, if not even more chimpanzee-like [13]. The attribution of OH 62 to *H*. *habilis* has been questioned, based on comparisons to *Au*. *sediba* [109], but regardless of its phylogenetic position, it provides evidence that increased upper limb loading, and by implication, frequent arboreal behavior, remained an important part of the locomotor behavior of at least some hominin lineages for millions of years after the first evidence for terrestrial bipedality [4–6].

Two earlier studies included some limited analyses of femoral and humeral diaphyseal cross sections of A.L. 288–1 taken at approximately midshaft [50, 51]. However, in each case the sections were derived from measurements of natural breaks on casts, supplemented with linear measurements on the original specimens, i.e., no radiography or CT imaging was performed. Although the humerus was included in one of these studies [50], there is no natural break near midshaft on either humerus of A.L. 288–1 (Fig 2), so it is not clear how this section was obtained. Furthermore, cross-sectional properties for A.L. 288–1 in this study [50] were calculated using an approximate eccentric ellipse model based on linear breadth measurements. It is not surprising, then, that cross-sectional properties in both studies ([50, 51] and Jungers, pers. comm.) vary by up to almost 30% from those obtained here (Table 1). The current properties, derived directly from micro-CT scans at well-defined locations, should be used in any future analyses.

A.L. 288–1's femur is also strong relative to either hip or knee articular size, as is the Stw 99 (*Au*. *africanus*) femur (relative to hip size). The overall greater robusticity of limb bone diaphyses in early hominins relative to modern humans has been previously noted [4]. The present study results indicate that this is specifically characteristic of australopiths and not to the same extent of early *Homo*. This difference implies a significant reduction in muscular loadings of both the humerus and femur in *H*. *erectus* relative to australopiths, if diaphyseal strength/articular size can be taken to represent relative muscular strength during life (S1 Text). This interpretation assumes that other factors that may influence relative articular size, including joint reaction force (relative to body size) and joint excursion [43, 58, 59], were similar between the groups compared. Although possibly somewhat variable in certain respects [110], joint excursion in the limbs is likely to have been relatively similar among all early hominins [111]. Joint reaction forces relative to body mass probably did vary, at least in the hip (see below). However, because of its much greater developmental plasticity, diaphyseal strength relative to articular size should still reflect in-vivo muscular loadings relative to joint reaction forces, including muscles not primarily involved in joint stabilization (such as the gluteal abductors).

If australopiths were indeed characterized by greater applied muscular forces in their limbs than *H*. *erectus* or modern humans, this observation has interesting implications for hominin evolutionary scenarios involving metabolic trade-offs between different organ systems [52–56, 112]. Muscular strength relative to body size is substantially greater in chimpanzees than in modern humans including even trained athletes (see S2 Text). The present results imply that this characteristic may also have been true of australopiths. There is evidence for marked evolutionary divergence between modern humans and chimpanzees in metabolite concentrations specifically in the brain and skeletal muscle [52]. Based on these results, the authors speculated that "While the molecular mechanism linking metabolic divergence with changes in muscular strength on the human evolutionary lineage cannot be determined based on our observations alone, we hypothesize that metabolic evolution of human muscle and brain metabolites may have occurred in parallel … Our results indicate that the reallocation of energy to energetically costly human brains may have required further decrease in energy expense in the skeletal muscle, at least during peak performance" ([52]: 6). Other authors have proposed similar scenarios based on comparative studies within vertebrates more broadly [53–55]. Recent work demonstrating a higher basal metabolic rate in modern humans than in great apes (as well as greater body fat storage) [113] indicates that certain aspects of these scenarios may need to be modified, i.e., humans may be able to afford a relatively larger brain in part because of our higher metabolic rate. However, other factors, including locomotor energetic efficiency, were also likely important [113]. Thus, the marked increase in relative brain size of early *Homo*, especially *H*. *erectus*, compared to australopiths [114] may have been facilitated in part by reductions in energy allocated to skeletal muscle.

If true, these observations could have other behavioral implications. For example, the capacity for increased peak power output of muscles in certain situations, such as defense against or escape from predators, or during inter-taxon aggression, may have had important selective advantages prior to the development of effective weapons [115]. The development of more efficient tools for food procurement or processing may also have decreased selective pressures for muscular strength. Increased muscular strength in extant great apes relative to modern humans is apparently attributable to a difference in specific force generated per volume of muscle tissue, rather than an increase in muscle mass in apes, although the cellular and molecular mechanisms involved have yet to be identified [56] (S2 Text). Thus, despite their implied greater muscular strength, australopiths may not necessarily have been characterized by large muscles relative to body mass.

As noted earlier, in terms of terrestrial locomotion, A.L. 288–1 (the best represented individual of *Au*. *afarensis*) shows many skeletal adaptations for bipedality [4]. Some aspects of Lucy’s proximal femoral morphology, however, indicate that her bipedal gait may have differed slightly from that of later *Homo*. A relatively small femoral head in australopiths in general has long been observed, although the mechanical significance of this observation has been debated [116–122]. One issue in this regard concerns which overall "size" parameter to use for evaluating relative femoral head size [51, 117, 120, 122]. The present analyses show that compared to modern humans or *H*. *erectus*, A.L. 288–1 has a small femoral head, but not a small knee, relative to distal humeral articular breadth. This observation suggests that overall lower limb mechanical loading was not reduced, i.e., that A.L. 288–1 was bipedal when on the ground, but that the joint reaction force on the femoral head was reduced relative to lower limb loadings as a whole (also see evidence for a relatively modern ankle joint and calcaneus in *Au*. *afarensis* [90, 91]). In contrast, a biomechanical model of the pelvis and proximal femur based on modern humans predicts that hip JRF would have been *increased* in A.L. 288–1 (see S3 Text, S2 Fig). It was hypothesized that she may have laterally deviated her body center of mass (COM), i.e., leaned over her stance limb, to reduce hip JRF during walking [51]. Note that this argument does not imply a developmentally plastic response to reduced hip joint loading, given the evidence for genetic constraint on articular size [39, 41, 57], but rather was more likely a long-term evolutionary (genetic) adaptation to reduced hip joint loadings in australopiths (and possibly other early hominins [5]) in conjunction with a different gait pattern.

The present new results for A.L. 288–1 are consistent with this hypothesis. Her superior and inferior femoral neck cortical breadths are asymmetric and more similar to modern humans than to nonhuman hominoids, but are also somewhat intermediate in asymmetry. This difference implies a more vertically oriented hip JRF than in modern humans, which would result from lateral deviation of the body COM during stance [14]. Lucy’s proximal femoral diaphysis is mediolaterally buttressed, but less so than in early *Homo* femora, even though her pelvic and femoral neck morphology would predict *greater* buttressing (S2 Fig). (Allometric scaling is unlikely as an explanation for this observation because large australopith specimens show the same pattern.) This observation is again consistent with a reduced hip JRF [51]. The morphology of other australopith femora is similar to that of A.L. 288–1, suggesting a similar gait modification.

If australopiths did indeed employ greater lateral deviation of the body COM over the stance limb during bipedal gait, it would likely have involved a substantial increase in metabolic cost of locomotion (S3 Text). Together with other morphological attributes, such as a relatively short hind limb [51, 65], this difference in turn may have limited long-distance terrestrial mobility in australopiths.

## Conclusions

Although bipedal when on the ground, the limb bone structural proportions of A.L. 288–1 provide evidence for substantially more arboreal, i.e., climbing behavior than either modern humans or *Homo erectus*. The frequency and magnitude of force required to stimulate bone modeling and remodeling of this kind [27] implies that this behavior was adaptively significant and not a trivial component of the locomotor repertoire [4, 89]. Possible reasons for using the trees more often include foraging for food and escape from predators. Furthermore, there is evidence that terrestrial bipedal gait in A.L. 288–1 may have differed in subtle but important ways from that of later *Homo*, decreasing locomotor efficiency when on the ground and limiting terrestrial mobility. Overall muscular strength relative to body size was likely greater than in *Homo*, perhaps reflecting less reliance on technology for food procurement/processing and defense. Where possible to evaluate, the same morphological attributes are present in other australopith specimens as well as *H*. *habilis sensu stricto*, i.e., OH 62 [13]. Overall these observations imply fundamental differences in ecology and behavior between australopiths and *Homo erectus*. It is likely that a number of different forms of terrestrial bipedality were practiced by early hominins, and that arboreal behavior remained an important part of the locomotor repertoire in particular taxa for millions of years.

## Supporting Information

S1 Fig Relative humeral diaphyseal strength and forearm muscle size. Regression of the residuals of humeral diaphyseal strength (polar section modulus) on humeral head superoinferior breadth against the residuals of maximum forearm muscle area on humeral head superoinferior breadth in modern 17-year-old human growth study participants (see S1 Text).
(TIF)

S2 Fig Effects of pelvic and proximal femoral morphology on hip joint reaction force and femoral shaft bending. Predicted abductor (M) and hip joint reaction (J) forces, and femoral diaphyseal bending moments during the stance phase of gait in a) modern humans, b) hypothetical early *Homo*, and c) A.L. 288–1. Force triangles in b) and c) show predicted vectors (dark) relative to modern humans (light). An increase in femoral neck length and biacetabular breadth (hypothetical in early *Homo* but consistent with other morphological evidence) leads to predicted increases in mediolateral bending of the proximal femoral shaft in both fossil taxa, larger in A.L. 288–1 than in early *Homo*, and an increase in hip joint reaction force in A.L. 288–1 relative to modern humans and early *Homo*. W: body weight (mass); W_L_: weight of lower limb; W_s_: superimposed body weight (W—W_L_); I: force in lateral tension band of knee. a) and b) from Ref. 1 in S3 Text, c) from Ref. 10 in S3 Text; see S3 Text for more discussion.
(TIF)

S1 Table CT scan parameters for A.L. 288–1. (XLSX)

S2 Table Reduced major axis statistics and comparisons between samples. (XLSX)

S3 Table Relative deviations of fossil specimens from modern humans and chimpanzees. (XLSX)

S4 Table Femoral biomechanical neck length and total length' in fossil specimens. (XLSX)

S5 Table Bilateral asymmetry in A.L. 288–1 right and left humeri. (XLSX)

S1 Text Diaphyseal Strength/Articular Size as a Proxy for Relative Muscular Strength. (DOCX)

S2 Text Muscle Strength in Chimpanzees. (DOCX)

S3 Text Hip Joint Loading and Gait Energetics in Modern Humans and Early Hominins. (DOCX)
