# Supplementary figures and images for: Limb Bone Structural Proportions and Locomotor Behavior in A.L. 288-1 ("Lucy")
Source: PLoS One. 2016 Nov 30;11(11):e0166095. doi: 10.1371/journal.pone.0166095 (PMC5130205; doi:10.1371/journal.pone.0166095)

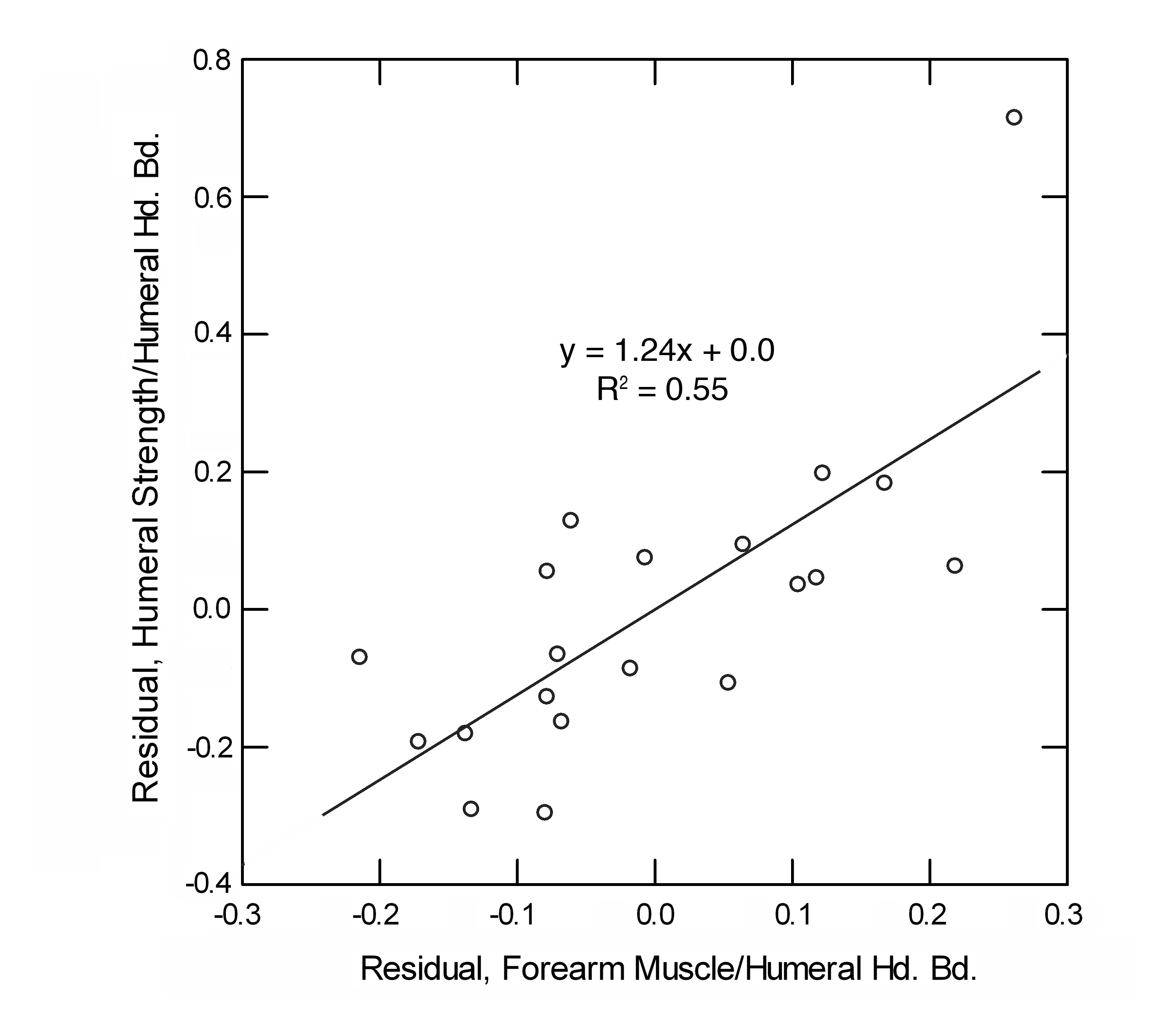

Supplement: S1 Fig — Regression of the residuals of humeral diaphyseal strength (polar section modulus) on humeral head superoinferior breadth against the residuals of maximum forearm muscle area on humeral head superoinferior breadth in modern 17-year-old human growth study participants (see S1 Text). (TIF) [file pone.0166095.s001.tif]

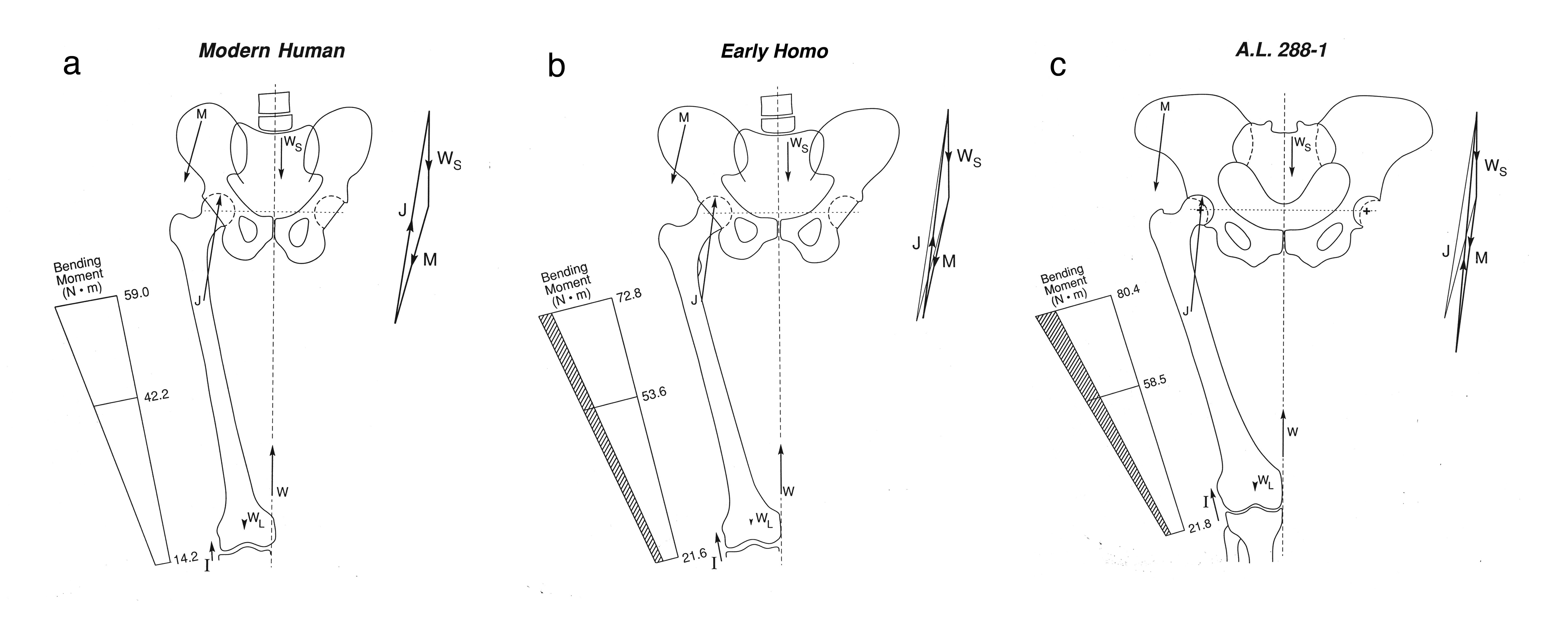

Supplement: S2 Fig — Predicted abductor (M) and hip joint reaction (J) forces, and femoral diaphyseal bending moments during the stance phase of gait in a) modern humans, b) hypothetical early Homo, and c) A.L. 288–1. Force triangles in b) and c) show predicted vectors (dark) relative to modern humans (light). An increase in femoral neck length and biacetabular breadth (hypothetical in early Homo but consistent with other morphological evidence) leads to predicted increases in mediolateral bending of the proximal femoral shaft in both fossil taxa, larger in A.L. 288–1 than in early Homo, and an increase in hip joint reaction force in A.L. 288–1 relative to modern humans and early Homo. W: body weight (mass); WL: weight of lower limb; Ws: superimposed body weight (W—WL); I: force in lateral tension band of knee. a) and b) from Ref. 1 in S3 Text, c) from Ref. 10 in S3 Text; see S3 Text for more discussion. (TIF) [file pone.0166095.s002.tif]
