# Supplementary material for: Limb Bone Structural Proportions and Locomotor Behavior in A.L. 288-1 ("Lucy")
Source: PLoS One. 2016 Nov 30;11(11):e0166095. doi: 10.1371/journal.pone.0166095 (PMC5130205; doi:10.1371/journal.pone.0166095)
Supplement: S1 Text — (DOCX) [file pone.0166095.s008.docx]

**S1 Text. Diaphyseal Strength/Articular Size as a Proxy for Relative Muscular Strength.**

In contrast to long bone diaphyseal cross-sectional geometry, which is developmentally plastic [1-4], long bone articular size is less directly affected by changes in mechanical loading during life [5-7]. This is clearly shown, for example, in studies of human upper limb bilateral asymmetry, in which diaphyseal cross-sectional dimensions track differences in limb use [8, 9], while articular dimensions do not [9, 10]. The same general morphological patterning has been shown recently in a nonhuman primate [11], although without accompanying behavioral data.

Many developmental studies of humans have demonstrated strong effects of muscular growth on long bone diaphyseal cross-sectional size or strength (adjusted for body size) [12]. In a previous study, growth velocities in long bone strength were found to be tightly correlated with changes in mechanical loadings, both gravitational and muscular (gravitational loadings were approximated by body mass $\cdot$ bone length, and muscular loadings by estimated cross-sectional muscle area) [13]. Gravitational effects predominated in the lower limb, while muscular effects were more significant in the upper limb, particularly among males. Articular properties were not included in that study, in part because it is difficult to measure articular size radiographically in younger juveniles. However, articular breadths are available for the older individuals in the study [14]. We focus here on the upper limb because it more clearly demonstrates the effects of muscular loadings while reducing the confounding effects of body mass variation. Individuals at 17 years of age - the oldest age group with complete data for all 20 subjects in the study - were analyzed. Linear regressions of log-transformed data were carried out for humeral diaphyseal strength and forearm muscle area against humeral head superoinferior breadth, and residuals calculated (see [13] for measurement details; arm muscle breadths were not available, but forearm and arm muscle areas are highly correlated in children [15].)

As shown in S1 Figure, there is a significant positive relationship between residual diaphyseal strength and residual muscle area, relative to joint size (r = .74, p < .001). The relationship is still significant within sex (r = .64-.67, p < .05, n = 10, both sexes). If residuals are calculated using body mass instead of articular breadth as the "size" variable, results are similar (r = .70, p = .001). Thus, in this sample diaphyseal strength relative to articular size reflects, to a large extent, relative muscle size. Note that while muscle size is used here as a proxy for muscle strength, it is quite possible that between evolutionarily divergent groups differences in muscle strength (and thus bone loadings) could result from other physiological mechanisms that do not involve muscle mass variation (see main text and S2 Text).

1. Ruff CB. Ontogenetic adaptation to bipedalism: Age changes in femoral to humeral length and strength proportions in humans, with a comparison to baboons. J Hum Evol. 2003; 45:317-49.

2. Ruff CB, Burgess ML, Bromage TG, Mudakikwa A, McFarlin SC. Ontogenetic changes in limb bone structural proportions in mountain gorillas (*Gorilla beringei beringei)*. J Hum Evol. 2013; 65(6):693-703. Epub 2013/10/17. doi: S0047-2484(13)00146-2 [pii] 10.1016/j.jhevol.2013.06.008. PubMed PMID: 24129040.

3. Ruff CB, Holt BH, Trinkaus E. Who's afraid of the big bad Wolff? Wolff's Law and bone functional adaptation. Am J Phys Anthropol. 2006; 129:484-98.

4. Sarringhaus LA, MacLatchy LM, Mitani JC. Long bone cross-sectional properties reflect changes in locomotor behavior in developing chimpanzees. Am J Phys Anthropol. 2016; 160:16-29. doi: 10.1002/ajpa.22930. PubMed PMID: 26780478.

5. Lieberman DE, Devlin MJ, Pearson OM. Articular area responses to mechanical loading: Effects of exercise, age, and skeletal location. Am J Phys Anthropol. 2001; 116:266-77.

6. Trinkaus E, Churchill SE, Ruff CB. Postcranial robusticity in *Homo*, II: Humeral bilateral asymmetry and bone plasticity. Am J Phys Anthropol. 1994; 93:1-34.

7. Ruff CB, Burgess ML, Junno JA, McFarlin SC, Mudakikwa A, Bromage TG, et al. Phylogenetic and environmental effects on limb bone structure in *Gorilla*. . Am J Phys Anthropol. 2016; 159 (S62):275.

8. Shaw CN. Is 'hand preference' coded in the hominin skeleton? An in-vivo study of bilateral morphological variation. J Hum Evol. 2011; 61(4):480-7. doi: S0047-2484(11)00156-4 [pii] 10.1016/j.jhevol.2011.06.004. PubMed PMID: 21839491.

9. Sládek V, Ruff CB, Berner M, Holt B, Niskanen M, Schuplerová E, et al. The impact of subsistence changes on humeral bilateral asymmetry in Terminal Pleistocene and Holocene Europe. J Hum Evol. 2016; 92:37-49.

10. Auerbach BM, Ruff CB. Limb bone bilateral asymmetry: variability and commonality among modern humans. J Hum Evol. 2006; 50:203-18.

11. Reeves NM, Auerbach BM, Sylvester AD. Fluctuating and directional asymmetry in the long bones of captive cotton-top tamarins (*Saguinus oedipus*). Am J Phys Anthropol. 2016; 160(1):41-51. doi: 10.1002/ajpa.22942. PubMed PMID: 26801822.

12. Kindler JM, Lewis RD, Hamrick MW. Skeletal muscle and pediatric bone development. Curr Opin Endocrinol Diabetes Obes. 2015; 22(6):467-74. doi: 10.1097/MED.0000000000000201. PubMed PMID: 26414082.

13. Ruff CB. Growth in bone strength, body size, and muscle size in a juvenile longitudinal sample. Bone. 2003; 33:317-29.

14. Ruff CB. Body size prediction from juvenile skeletal remains. Am J Phys Anthropol. 2007; 133(1):698-716. PubMed PMID: 17295297.

15. Boye KR, Dimitriou T, Manz F, Schoenau E, Neu C, Wudy S, et al. Anthropometric assessment of muscularity during growth: estimating fat-free mass with 2 skinfold-thickness measurements is superior to measuring midupper arm muscle area in healthy prepubertal children. Am J Clin Nutr. 2002; 76(3):628-32. PubMed PMID: 12198010.
