# Supplementary material for: Limb Bone Structural Proportions and Locomotor Behavior in A.L. 288-1 ("Lucy")
Source: PLoS One. 2016 Nov 30;11(11):e0166095. doi: 10.1371/journal.pone.0166095 (PMC5130205; doi:10.1371/journal.pone.0166095)
Supplement: S2 Text — (DOCX) [file pone.0166095.s009.docx]

**S2 Text. Muscle Strength in Chimpanzees.**

Studies comparing muscular strength in chimpanzees and humans have produced somewhat variable results, although in all cases chimpanzees were stronger than humans relative to body mass. Early studies of the maximal forelimb strength of two captive chimpanzees engaged in a pulling task documented approximately 4X the strength per body mass of well-conditioned young adult human males [1, 2]. Without providing supporting data, the author considered the difference to be due to greater strength per "muscle girth". A later study using a similar experimental set-up found a smaller difference in muscular strength per body mass between male humans and chimpanzees averaging 36% [3]. The author attributed part of the difference in results from the earlier study to the effect of emotional state (one chimpanzee in the earlier study had been "enraged" while pulling), which suggests the involvement of neurological factors. In another study, an elbow flexion task, carried out on five chimpanzees (three males, four juveniles), produced 2.5X greater strength per body mass than in seven adult human males in good physical condition, including one who was a weight-lifter [4]. Strength per calculated forelimb muscle girth was still 41% greater in the chimpanzees. Five adult or subadult chimpanzees produced about 2X the force per body mass of an age, sex, and ethnically mixed sample of 41 humans in a pulling task involving both the upper and lower limbs [5]. All of these studies were potentially influenced by the expected greater upper limb strength of chimpanzees, although in at least some cases there was an attempt to factor in differences in muscle area.

In the only study to date focusing on the lower limb, where humans might be expected to have a strength advantage, bonobos still exhibited about twice the work output of humans per estimated muscle cross-sectional area [6]. The authors concluded that the most likely explanation was a species difference in intrinsic muscular structure or control. However, in another study, no difference in muscle fiber-type composition was found in comparisons between chimpanzees and humans [5], suggesting that neural control mechanisms may be more likely. Neural adaptations have in fact been suggested as important in mediating training effects on muscle strength [7]. It should be noted that in all of these studies chimpanzees were captive, and in many cases apparently did not have the opportunity to exercise regularly, while many of the human subjects were recreational athletes. Thus, the results may be conservative given expected exercise training effects on muscular strength [7].

Another possible contribution to the relatively stronger long bone diaphyses of chimpanzees compared to modern humans is longer muscle moment arms in chimpanzee forelimbs and hind limbs, and thus greater bending under the same applied muscle forces. However, taken as a whole, limb muscle moment arms scaled to body mass are similar or variably greater in chimpanzees or humans, i.e., there is no overall increase in moment arms in chimpanzees [8]. Thus, increased muscular strength is a more likely explanation.

1. Bauman JE. The Strength of the chimpanzee and orang. Sci Mon. 1923; 16:432-40. PubMed PMID: WOS:000206561700012.

2. Bauman JE. Observations on the strength of the chimpanzee and its implications. J Mammalogy. 1926; 7:1-9.

3. Finch G. The bodily strength of the chimpanzee. J Mammal. 1943; 24:224-8.

4. Edwards WE, Clarke TE. Study of monkey, ape, and human morphology and physiology relating to strength and endurance. Phase IX: The strength testing of five chimpanzee and seven human subjects. Holloman Air Force Base, New Mexico: 6571st Aeromedical Research Laboratory Pub. ARL-TR-65-15; 1965.

5. Bozek K, Wei Y, Yan Z, Liu X, Xiong J, Sugimoto M, et al. Exceptional evolutionary divergence of human muscle and brain metabolomes parallels human cognitive and physical uniqueness. PLoS biology. 2014; 12(5):e1001871. doi: 10.1371/journal.pbio.1001871. PubMed PMID: 24866127; PubMed Central PMCID: PMC4035273.

6. Scholz MN, D'Aout K, Bobbert MF, Aerts P. Vertical jumping performance of bonobo (Pan paniscus) suggests superior muscle properties. Proc Biol Sci. 2006; 273(1598):2177-84. doi: 10.1098/rspb.2006.3568. PubMed PMID: 16901837; PubMed Central PMCID: PMC1635523.

7. Enoka RM. Muscle strength and its development - new perspectives. Sports Med. 1988; 6(3):146-68. doi: Doi 10.2165/00007256-198806030-00003. PubMed PMID: WOS:A1988Q193200003.

8. Thorpe SK, Crompton RH, Gunther MM, Ker RF, McNeill Alexander R. Dimensions and moment arms of the hind- and forelimb muscles of common chimpanzees (*Pan troglodytes*). Am J Phys Anthropol. 1999; 110(2):179-99. PubMed PMID: 10502242.
