# Supplementary material for: Limb Bone Structural Proportions and Locomotor Behavior in A.L. 288-1 ("Lucy")
Source: PLoS One. 2016 Nov 30;11(11):e0166095. doi: 10.1371/journal.pone.0166095 (PMC5130205; doi:10.1371/journal.pone.0166095)
Supplement: S3 Text — (DOCX) [file pone.0166095.s010.docx]

**S3 Text. Hip Joint Loading and Gait Energetics in Modern Humans and Early Hominins.**

A general model of pelvic-femoral mechanical loading is shown in S2a Figure [1]. The model is based on an experimental static analysis in one-legged stance [2], and is supported by much in-vivo data and clinical experience. It has been successfully employed in many orthopaedic studies to predict clinical outcomes of altered geometric relationships between abductor muscular and body weight moment arms about the hip, including variation in hip joint reaction forces (JRF) [3, 4]. Predicted magnitudes and orientations of hip JRFs using this model are also consistent with those measured in-vivo using instrumented prostheses [5]. The same principles have been used in finite element models to assess hip arthroplasty failure and femoral neck stress fracture risk [6, 7]. Mediolateral bending of the proximal femoral diaphysis under loading has been confirmed using in-vivo strain gauges, with bending strains increasing with a longer effective femoral neck length [8], again as predicted from the model (S2 Fig).

Both a wider biacetabular breadth and longer femoral neck appear to characterize all early hominins [9], and are consistent with the increased M-L bending strength of the proximal femur also observed in these taxa (S2b,c Fig). According to the model, though, A.L. 288-1 should exhibit more M-L strengthening of this region than early *Homo* (S2c Fig), yet the opposite is found (Fig 4). A reduction in hip JRF in A.L. 288-1, and australopiths generally, would lead to lower predicted increases in M-L bending loads in the proximal femur [10], which is consistent with their morphology in this region. Such a reduction could have been achieved through greater lateral deviation of the body center of mass (COM) over the stance limb [9, 10].

A recent kinematic study questioned the applicability of this traditional model to gait dynamics, showing using inverse dynamics that the body weight moment arm about the hip can vary from that predicted from biacetabular (inter-hip) breadth [11]. However, the effective mechanical advantage (EMA) of the gluteal abductors (abductor moment arm/body weight moment arm) was actually significantly correlated between the dynamic measurement and the traditional measurement in this study (for walking, r = 0.722, p < .001 (n = 25) using femoral neck length and half biacetabular breadth in the traditional model, our calculations from their data). This indicates that the static model shown in S2a Figure still has validity for estimating abductor force, and by implication hip JRF during stance.

Because abductor force constitutes only a minor percentage of total energetic output during normal gait (in straight-line, level walking) [11, 12], changes in the EMA of the abductors, within normal limits, have little effect on locomotor cost [11]. However, based on clinical studies, major alterations in positioning of the body COM over the hip joint, as argued here for A.L. 288-1, would probably have had significant effects on locomotor energetics. Studies of subjects with exaggerated lateral deviation of the body COM during gait due to compromised gluteal abductor function show marked increases in cost of transport. Many of these studies have been carried out on individuals with spinal cord injuries resulting from myelomeningocele (spina bifida), which affects lower limb muscular function to varying degrees. Lesions above the L4 spinal level (thus affecting the gluteal abductors) result in a gait pattern characterized by elevation of the swing side limb accompanied by lateral flexion of the trunk over the stance side, positioning the COM over the stance limb during walking [13-15]. Cost of transport is more than twice that of normal individuals, with a large portion of the increase attributable to these compensatory motions (as deduced from comparisons with costs associated with lower spinal injuries not affecting the gluteal abductors) [15-17]. In line with this, providing external lateral stabilization to patients with incomplete spinal cord injuries significantly reduces cost of transport [18].

None of these specific gait abnormalities would have characterized early hominins, of course. But if A.L. 288-1 (and other australopiths) walked with much greater lateral deviation of the COM than is typical for normal modern humans, this would have entailed a significant metabolic cost. Note that this inference is not based on relative pelvic breadth alone, which has been shown not to affect locomotor cost (at least in modern, relatively narrow-bodied humans) [11], but rather on the total morphological pattern of australopiths, including hip joint size and femoral neck and proximal diaphyseal morphology. While some studies have questioned the association between strain magnitudes and diaphyseal cortical bone remodeling [19], a large number of other experimental and observational studies support this inference [20-23].

1. Ruff CB. Biomechanics of the hip and birth in early *Homo*. Am J Phys Anthropol. 1995; 98:527-74.

2. McLeish RD, Charnley J. Abduction forces in the one-legged stance. J Biomechanics. 1970; 3:191-209.

3. Hurwitz DE, Andriacchi TP. Biomechanics of the hip. In: Rosenberg AG, Callaghan JJ, editors. The Adult Hip. Philadelphia: Lippincott-Raven; 1998. p. 75-85.

4. Skeikhzadeh A, Kendall O, Frankel V. Biomechanics of the hip. Basic Biomechanics of the Musculoskeletal System. Philadelphia: Wolters Kluwer Health; 2012. p. 206-23.

5. Bergmann G, Deuretzbacher G, Heller M, Graichen F, Rohlmann A, Strauss J, et al. Hip contact forces and gait patterns from routine activities. J Biomech. 2001; 34(7):859-71. PubMed PMID: 11410170.

6. Schwarzkopt R, Dong NNG, Fetto JF. Finite element analysis of femoral neck stress in relation to pelvic width. Bull NYU Hosp Jt Dis. 2011; 69(4):292-7.

7. Voo L, Armand M, Kleinberger M. Stress fracture risk analysis of the human femur based on computational biomechanics. Johns Hopkins APL Tech Digest. 2004; 25(3):223-30.

8. Aamodt A, Lund-larsen J, Eine J, Anderseon E, Benum P, Husby OS. *In vivo* measurements show tensile axial strain in the proximal lateral aspect of the human femur. J Orthop Res. 1997; 15:927-31.

9. Ruff CB, Higgins R. Femoral neck structure and function in early hominins. Am J Phys Anthropol. 2013; 150(4):512-25. Epub 2013/01/24. doi: 10.1002/ajpa.22214. PubMed PMID: 23341246.

10. Ruff CB. Evolution of the hominid hip. In: Strasser E, Rosenberger A, McHenry H, Fleagle J, editors. Primate Locomotion: Recent Advances. Davis, CA: Plenum Press; 1998. p. 449-69.

11. Warrener AG, Lewton KL, Pontzer H, Lieberman DE. A wider pelvis does not increase locomotor cost in humans, with implications for the evolution of childbirth. Plos One. 2015; 10(3):e0118903. doi: 10.1371/journal.pone.0118903. PubMed PMID: 25760381; PubMed Central PMCID: PMC4356512.

12. Donelan JM, Shipman DW, Kram R, Kuo AD. Mechanical and metabolic requirements for active lateral stabilization in human walking. J Biomech. 2004; 37(6):827-35. doi: 10.1016/j.jbiomech.2003.06.002. PubMed PMID: 15111070.

13. Duffy CM, Hill AE, Cosgrove AP, Corry IS, Mollan RAB, Graham HK. Three-dimensional gait analysis in spina bifida. J Pediatr Orthoped. 1996; 16(6):786-91. PubMed PMID: ISI:A1996VM99400016.

14. Gutierrez EM, Bartonek A, Haglund-Akerlind Y, Saraste H. Centre of mass motion during gait in persons with myelomeningocele. Gait Posture. 2003; 18(2):37-46. PubMed PMID: 14654206.

15. Duffy CM, Hill AE, Cosgrove AP, Corry IS, Graham HK. The influence of abductor weakness on gait in spina bifida. Gait & Posture. 1996; 4:34-8.

16. Bare A, Vankoski SJ, Dias L, Danduran M, Boas S. Independent ambulators with high sacral myelomeningocele: the relation between walking kinematics and energy consumption. Dev Med Child Neurol. 2001; 43(1):16-21. PubMed PMID: 11201417.

17. Duffy CM, Hill AE, Cosgrove AP, Corry IS, Graham HK. Energy consumption in children with spina bifida and cerebral palsy: A comparative study. Dev Med Child Neurol. 1996; 38(3):238-43. PubMed PMID: ISI:A1996UA93200006.

18. Matsubara JH, Wu M, Gordon KE. Metabolic cost of lateral stabilization during walking in people with incomplete spinal cord injury. Gait & Posture. 2015; 41:646-51.

19. Wallace IJ, Demes B, Mongle C, Pearson OM, Polk JD, Lieberman DE. Exercise-induced bone formation is poorly linked to local strain magnitude in the sheep tibia. Plos One. 2014; 9(6):e99108. doi: 10.1371/journal.pone.0099108. PubMed PMID: 24897411; PubMed Central PMCID: PMC4045900.

20. Macdonald HM, Cooper DM, McKay HA. Anterior-posterior bending strength at the tibial shaft increases with physical activity in boys: evidence for non-uniform geometric adaptation. Osteoporos Int. 2009; 20(1):61-70. PubMed PMID: 18496638.

21. Rantalainen T, Nikander R, Heinonen A, Suominen H, Sievanen H. Direction-specific diaphyseal geometry and mineral mass distribution of tibia and fibula: a pQCT study of female athletes representing different exercise loading types. Calcified tissue international. 2010; 86(6):447-54. doi: 10.1007/s00223-010-9358-z. PubMed PMID: 20383493.

22. Ruff CB, Holt BH, Trinkaus E. Who's afraid of the big bad Wolff? Wolff's Law and bone functional adaptation. Am J Phys Anthropol. 2006; 129:484-98.

23. Shaw CN, Stock JT. Habitual throwing and swimming correspond with upper limb diaphyseal strength and shape in modern human athletes. Am J Phys Anthropol. 2009; 140(1):160-72. Epub 2009/04/10. doi: 10.1002/ajpa.21063. PubMed PMID: 19358297.
